# Baseline MxA mRNA Expression Predicts Interferon Beta Response in Multiple Sclerosis Patients

**DOI:** 10.1371/journal.pone.0112758

**Published:** 2014-11-14

**Authors:** Elisabet Matas, Laura Bau, María Martínez-Iniesta, Lucía Romero-Pinel, M. Alba Mañé, Álvaro Cobo-Calvo, Sergio Martínez-Yélamos

**Affiliations:** 1 Multiple Sclerosis Unit, Neurology Department, Hospital Universitari de Bellvitge-IDIBELL, L'Hospitalet de Llobregat, Barcelona, Spain; 2 Translational Research Laboratory, Institut Català d'Oncologia- IDIBELL, L'Hospitalet de Llobregat, Barcelona, Spain; 3 Department of Neurology, Joan XXIII University Hospital, Universitat Rovira I Virgili, Tarragona, Spain; University of Sydney, Australia

## Abstract

**Background:**

Myxovirus resistance protein A (MxA) is a molecule induced after interferon-beta injection, mostly used to evaluate its bioactivity. There is little available data on clinical utility of baseline MxA mRNA status. The objective of the study is to investigate whether baseline MxA mRNA expression can predict relapse and disease progression in multiple sclerosis patients treated with interferon-beta.

**Methods:**

Baseline blood samples were obtained before the first interferon-beta dose was administered to evaluate MxA mRNA expression using real-time polymerase chain reaction (PCR). Demographic and clinical variables were prospectively recorded to define treatment responder and non responder groups.

**Results:**

104 patients were included in the study. Baseline MxA mRNA expression was significantly lower in the group of patients who met the definition of responders (1.07 vs 1.95, Student *t* test, p<0.0001). A threshold of 1.096 was established using Receiver Operating Characteristic analysis to differentiate between responders and non-responders (sensitivity 73.9%, specificity 69.0%). Survival analysis using this threshold showed that time to next relapse (p<0.0001) and to EDSS progression (p = 0.01) were significantly higher in patients with lower MxA titers.

**Conclusion:**

The results suggest that baseline MxA mRNA levels may be useful for predicting whether multiple sclerosis patients will respond or not to interferon-beta treatment.

## Introduction

Multiple sclerosis (MS) is an immune-mediated demyelinating disease of the central nervous system that mainly affects middle-aged adults and is a common cause of disability. Several therapies are available for the treatment of MS. Interferon beta (IFN-β) was the first approved and is one of the most common immunomodulatory therapies used for this condition. Results from clinical trials have shown a reduction in the MS relapse rate of about 30% with this agent [1]–[6]. Unfortunately, not all patients respond properly to MS therapies. A percentage of patients do not respond to treatment, and this fact could only be recognized after months or years of therapy. It would be of value being able to determine whether a patient will respond to each type of treatment so that the most appropriate therapy can be given before the disease relapses or progresses.

Myxovirus resistance protein A (MxA) is a molecule induced after injection of IFN-β, and its quantification could be considered a biomarker of IFN-β bioactivity [7]. There is little available data on MxA mRNA baseline status or its potential usefulness for indicating IFN-β treatment response. The objective of this study is to investigate whether MxA mRNA baseline expression has a role in predicting the occurrence of relapses or disease progression in MS patients treated with IFN-β.

## Methods

### Study Design

A prospective, observational, open-label, non-randomized study was performed in the Multiple Sclerosis Unit of Hospital Universitari de Bellvitge. Our MS clinic is the reference center for demyelinating diseases in the health district of *Gerència Territorial Barcelona Metropolitana Sud* in Catalonia, a region in the northeast of Spain.

### Ethics Statement

The study was approved by the Ethics Committee of Hospital Universitari de Bellvitge, and written informed consent to participate was obtained from each patient and control.

### Patients

Patient enrollment began in February 2008 and was completed in March 2011. Patients meeting the following inclusion criteria were prospectively selected: treatment-naïve, relapsing-remitting MS patients fulfilling the 2005 revised McDonald criteria [8] and achieving criteria to start IFN-β treatment. After selection, patients initiated treatment with one of three IFN-β products: IFN-β 1a 30 µg by intramuscular administration once weekly (Avonex), IFN-β 1a 44 µg subcutaneously three times weekly (Rebif44), or IFN-β 1b 8 million IU subcutaneously every other day (Betaferon/Extavia). Patients were not randomized to treatment. Therapy for each case was selected according to guidelines for MS and the standard medical practice in our center. Prospective follow-up was completed in May 2012. Treatment changes were not allowed during the study. For the development of survival curves, follow-up was finished when a relapse or increase in the EDSS score occurred. Patients that finished the follow-up before any of these events occurred or stopped the treatment, were censored for that analysis.

A cohort of non-MS controls was selected to perform gene standard curves (MxA and GAPDH) and to normalize MS patient samples.

Clinical assessment including the Expanded Disability Status Scale (EDSS) [9] was performed every 6 months following the start of treatment and at the time of relapse. Clinical, demographic and radiological data were recorded prospectively using the European Database for Multiple Sclerosis (EDMUS) [10]. Relapses were established based on the development of a new symptom or worsening of an old symptom attributable to MS, accompanied by consistent neurological dysfunction lasting at least 24 hours in the absence of fever and preceded by stability or improvement for at least 30 days [11]. EDSS progression was defined as an increase of at least 1 point on the EDSS score. The EDSS score had to be confirmed at least 6 months later to be defined as irreversible [12].

Treatment responders (R) and non-responders (NR) were defined as follows: Responders were patients presenting no relapses or EDSS progression during follow-up. Non-responders were those presenting relapses and/or EDSS progression. Two groups were defined in the non-responders group: Relapse-NR were patients presenting relapses but no increase on the EDSS score and EDSS-NR comprised of patients presenting EDSS progression or relapses and additionally EDSS progression at follow-up.

### Samples

Blood samples were obtained before the first IFN-β dose and after 12 months of treatment, in the absence of signs of infection or corticosteroid treatment for relapse. Ten milliliters of peripheral blood from MS patients and controls was collected in an EDTA tube. Mononuclear cells were separated on a Ficoll-Hypaque density gradient. RNA extraction was performed with Ultraspec-II RNA isolation system (Biotecx Laboratories, Texas, USA) following the manufacturer's instructions.

Complementary DNA was prepared by reverse transcription using M-MLV Reverse Transcriptase (Invitrogen Life Technologies, Carlsbad, USA) according to the manufacturer's recommendations. Real-time PCR was performed on a Light Cycler 480 system (Roche, Mannheim, Germany) using the Lightcycler 480 sybr green master kit (Roche, Mannheim, Germany). Results were normalized to the expression level of the glyceraldehyde-3-phosphate dehydrogenase (GAPDH) housekeeping gene to avoid differences due to possible RNA degradation or variable reverse transcription efficiency. Primers for MxA and GAPDH were designed following the description provided by other authors [13]. Standard curves were performed for each primer using control samples diluted to different concentrations. A sample from these curves was used as a standard and run in each experiment. Results obtained were normalized to a calibrator. A pool of healthy control samples was used as a calibrator and run during each PCR assay. MxA and GAPDH PCR quantities were determined using these standard curves and were normalized to GAPDH and to a calibrator. Results were expressed as MxA mRNA expression levels relative to GAPDH expression levels.

### Statistical analysis

Differences in baseline clinical characteristics were analyzed using the Student *t* test, chi-square test, or Mann-Whitney *U* test, as appropriate. Baseline MxA mRNA expression in the R and NR groups was compared using the Student *t* test. The optimal cut off value for MxA expression was determined using receiver operating characteristics (ROC) analysis. Kaplan Meier survival curves were carried out to study time to the next relapse and to progression of disability. Results obtained from baseline samples were used to establish the cut-off to determine the presence of MxA mRNA induction at month 12. The threshold was defined as mean + 3SD. [14]-[16] Kaplan Meier survival curves were performed to study differences in time to the next relapse and in time to progression of disability between MxA induced and non induced patients.

All statistical analyses were performed with the Statistical Package for the Social Sciences, 20.0 (SPSS Inc., Chicago, USA). A *P* value of <0.05 was considered statistically significant for the comparisons.

## Results

### Patients

A total of 104 relapsing-remitting MS patients were included in the study. The inclusion period was completed in March 2011 and follow-up in May 2012. The patients' baseline clinical and radiological characteristics are shown in Table 1. No statistical differences regarding these characteristics were found.

**Table 1:** Clinical and demographic baseline characteristics.

	High-MxA (MXA >1.069)	Low-MxA (MXA <1.069)	*p*	TOTAL
**Patients**	52	52	-	104
**Sex, n(%) female**	39 (75.0%)	36 (69.2%)	0.51^a^	75 (72.1%)
**Age at onset, years, mean (SD)**	32.31 (8.26)	33.77 (8.41)	0.37^b^	33.04 (8.33)
**Relapses pretreatment, mean (SD)**	2.37 (1.44)	2.50 (1.61)	0.65^b^	2.43 (1.52)
**Duration MS pretreatment, years, median (IQR)**	1.53 (0.82-3.19)	1.40 (0.75–3.52)	0.59^c^	1.53 (0.78–3.19)
**Initial EDSS score, median (IQR)**	1.5 (1.0–2.0)	1.5 (1.0–2.0)	0.99^c^	1.5 (1.0–2.0)
**Duration of follow-up, years, median (IQR)**	1.98 (1.52–2.58)	1.74 (1.04–2.90)	0.55^c^	1.94 (1.09–2.64)
**Baseline MRI**				
**-Gadolinium enhancement**	16/42 (38.1%)	17/42 (40.5%)	0. 82^a^	33/84 (39.3%)
**-Infratentorial lesions**	37/49 (75.5%)	35/49 (71.4%)	0.65^a^	72/98 (73.4%)
**Interferon, n (%)**				
**-Rebif44**	26 (50.0%)	27 (51.9%)		53 (51.0%)
**-Betaferon/Extavia**	22 (42.3%)	20 (47.6%)	0.89^a^	42 (40.4%)
**-Avonex**	4 (7.7%)	5 (9.6%)		9 (8.9%)

a Chi-square test.

b Student *t*-test.

c Mann-Whitney *U* test.

Abbreviations: EDSS: Expanded Disability Scale; IQR: interquartile range; SD: standard deviation.

### Responders and non-responders

At the end of follow-up, 58 (55.8%) patients met the definition of responders and 46 (44.2%) patients were classified as non-responders. The 46 patients in the NR group were Relapse-NR and 16 (15.3%) EDSS-NR. None of the patients developed a secondary progressive MS during the study, thus the 16 patients in the EDSS-NR group with confirmed EDSS progression also had relapses.

### Baseline MxA expression

The mean baseline MxA mRNA expression level was significantly higher in the NR group (1.95, SD 1.32) than in the R group (1.07, SD 0.86) (Student *t* test, p<0.0001). ROC analysis was performed to establish a cut-off value for MxA mRNA expression that could differentiate between R and NR. A cut-off of 1.096 yielded the best sensitivity (73.9%) and specificity (69.0%) values (area under the curve  = 0.732). The positive predictive value was 76.9% and the negative predictive value, 65.4%.

Patients were then classified as high-MxA or low-MxA according to whether MxA mRNA levels were above or below 1.096. The baseline characteristics of the high-MxA and low-MxA groups were analyzed, and no statistical differences were found. (Table 1) Mean relapse rate during the study was 1.23 (SD 1.28) in the high-MxA group and 0.46 (SD 0.99) in the low-MxA group, (Student *t* test, p = 0.001). There were no differences in the duration of study follow-up between the two groups (years, median 1.98, IQR 1.52–2.58 *vs* 1.74, IQR 1.04–2.90, respectively) (Mann-Whitney *U* test, p = 0.554).

Fourteen patients abandoned or changed treatment before a relapse or EDSS progression occurred: 9 interrupted treatment for pregnancy intention, 3 switched treatment because of adverse events (2 intense flu-like symptoms, 1 persistent lymphopenia), 1 had problems with the injector handling, and 1 abandoned therapeutic intervention. Median follow-up in the study for these patients was 1.29 years (IQR 0.78–1.91). There were no differences between the groups regarding the number of patients who did not complete follow-up (6 high-MxA and 8 low-MxA, chi-square test p = 0.33).

### Survival analysis

**Figure 1:**
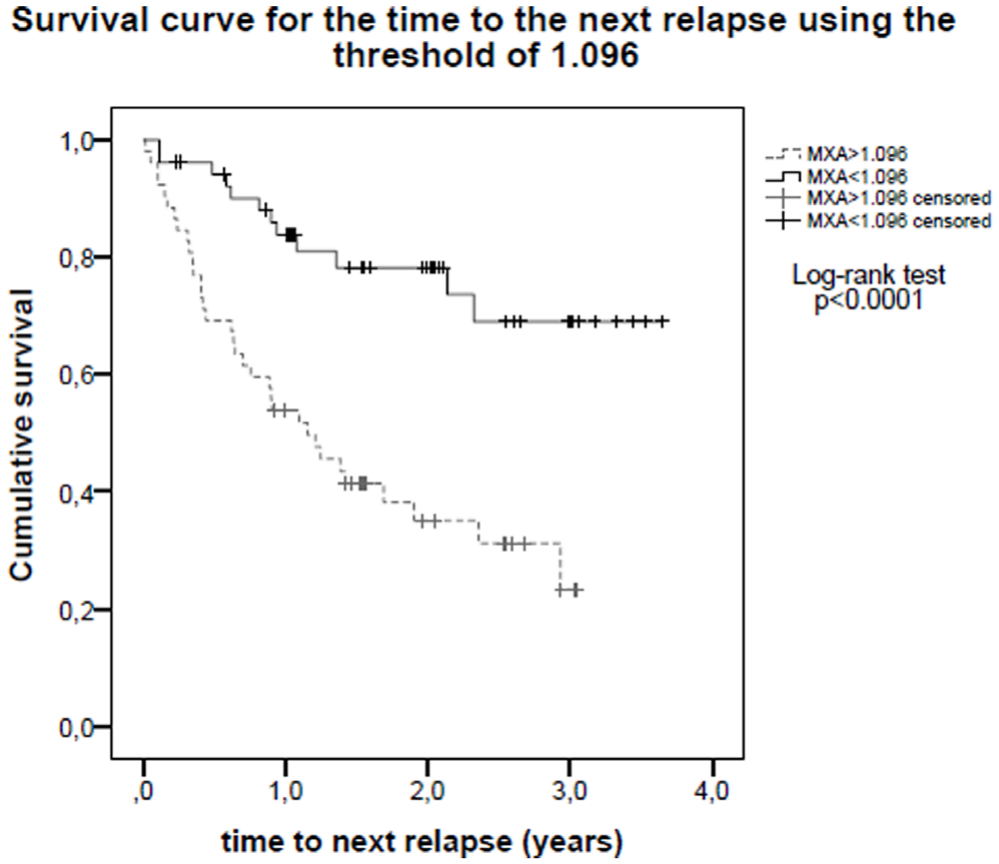
Survival curve for the time to the next relapse using the 1.096 threshold. Patients belonging to the low-MxA group (MxA <1.096) showed a significantly longer time to the next relapse (p<0.0001).

**Figure 2:**
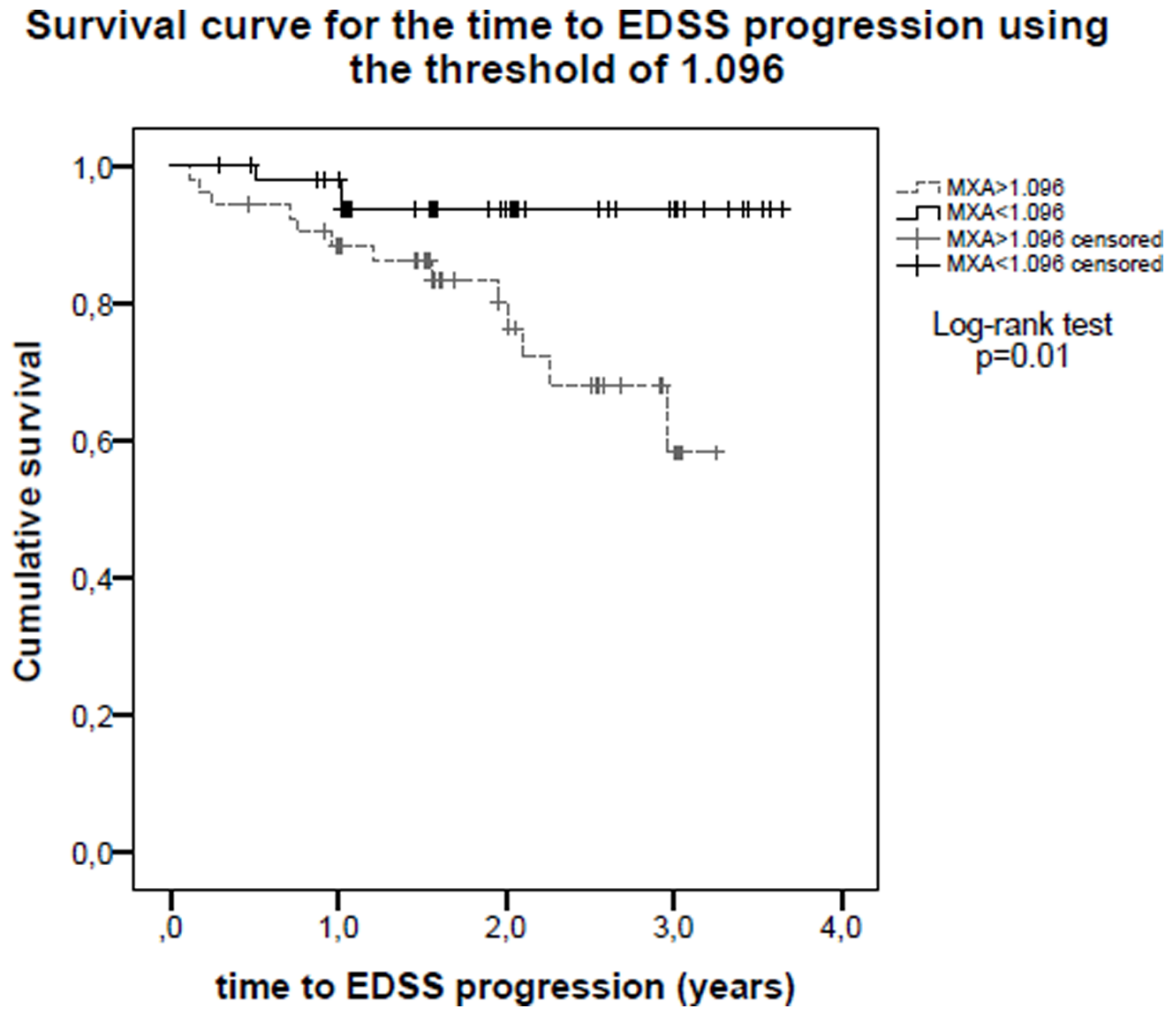
Survival curve for the time to EDSS progression using the 1.096 threshold. Patients belonging to the low-MxA group (MxA <1.096) showed a significantly longer time to increase by one point on the EDSS scale (p = 0.01).

Survival analysis for relapses (Fig. 1) and EDSS progression (Fig. 2) was performed using the 1.096 threshold. In the low MxA group, the time to the next relapse and to increase one point on the EDSS scale confirmed at 6 months was significantly longer compared with the high MxA group (25% of patients experienced the next relapse (percentile 75) in 2.14 years in the low-MxA group vs 0.40 years in the high-MxA group, log-rank p<0.0001)(25% of patients experienced EDSS progression (percentile 75) in undefined time in low-MxA group vs 2.09 years in the high-MxA group, log-rank p = 0.01).

### MxA induction

Evaluation of MxA mRNA expression after 12 months of treatment was performed in 96 patients. Eighteen patients showed absence of MxA induction (defined as levels below baseline MxA mRNA mean (1.46) +3SD (1.17) [14]–[16]) and in the remaining 78 patients MxA induction was demonstrated. No statistical differences were detected between high-MxA and low-MxA groups at baseline in the proportion of patients with or without MxA induction after one year of treatment (8 patients without MxA induction (16.7%) and 40 with MxA induction (83.3%) after one year of treatment in the high-MxA group at baseline vs 10 patients without induction (20.8%) and 38 with induction (79.2%) in the low-MxA group at baseline, *Chi square* test p = 0.60).

Survival analyses were performed to study differences in time to the next relapse and to progression of disability between MxA induced and non-induced patients at month 12. No statistical differences were found between both groups (median time to next relapse, MxA induced 2.93 years vs MxA non-induced 2.32 years, log rank p = 0.72; median time to EDSS progression, MxA induced undefined time vs MxA non-induced undefined time, log rank p = 0.21).

## Discussion

Approximately 30% to 50% of MS patients do not respond to IFN-β treatment [12], [17]. Two main types of mechanisms in the failure to respond to IFN-β treatment have been described [18]. The first one is in part a consequence of differing baseline characteristics, leading to interindividual differences in the response to this drug. The baseline characteristics of IFN-induced gene expression that conform to the so-called IFN signature [18]–[20], are factors that could explain these differences. High endogenous expression of these genes has been demonstrated in a subgroup of patients, referred to as IFN-^high^. Several authors have studied the implications of the IFN signature in the IFN response. The results suggest that when the type I IFN pathway is fully activated at baseline (IFN-^high^), there is a lack of pharmacological effect due to a loss of the ability for further stimulation by IFN-β administration [19], [21], [22]. The IFN signature, and therefore the MxA as one of the genes constituting the IFN signature [19], [20], could have a potential role as a biomarker of the IFN-β response.

A second mechanism in the failure to respond to IFN-β therapy is probably due to immunogenic factors leading to a decrease in the bioavailability of IFN-β. Development of NAbs is one of the main types but also others as the presence of different IFN receptor isoforms could contribute to this decrease [23]. Independently of the mechanism involved, a reduction of the expression of induced interferon responsive genes is detected including the MxA expression.

The present study evaluates if baseline MxA could have a role in predicting the IFN-β response. The results suggest that baseline MxA mRNA status can predict whether patients will respond or not to IFN-β before starting treatment. Previously published results suggest that patients with a less activated endogenous type I IFN pathway would have greater ability to up-regulate genes levels after the start of IFN, which would lead to a favorable response [19], [20]. We hypothesize that patients with low baseline MxA status, as one of the genes involved in the IFN signature, would up-regulate MxA levels when IFN is started and therefore would have a favorable response. On the other hand, high MxA titers at baseline would indicate an innate activation of genes related to IFN response and a less inducible pathway, which would result in failure to respond to IFN-β treatment.

Baseline MxA expression has been evaluated in several studies. Some authors have suggested that spontaneous MxA mRNA levels in MS patients may be useful to identify patients with active disease forms [24] and those experiencing a relapse [25].They found that higher baseline MxA mRNA levels are related to a longer time to a new relapse. Essential differences were found between these studies and the present. The objective of our study was to evaluate if baseline MxA expression has a role in predicting the response to IFN-β treatment while in Van der Voort study [26] the main objective was to evaluate if MxA is related to clinical disease activity in early MS untreated patients. Since the objectives are different, the populations included were also different: in our study, a homogeneous cohort of treatment naïve relapsing-remitting MS patients with at least one relapse in the previous year and achieving criteria to start treatment was selected to evaluate clinical response after beginning IFN-β treatment. In van der Voort study, patients presenting with a clinically isolated syndrome suggestive of MS or recently diagnosed with relapsing-remitting MS were recruited. In the subgroup of 50 patients that started treatment with INF-β, no differences in baseline MxA mRNA levels were found between responders and non-responders, probably due to the small sample of patients as themselves suggest. It could be possible that untreated MS patients with low baseline MxA levels would have more chance to experience a relapse earlier and, at the same time, would have greater ability to induce MxA and therefore to show a better response to the treatment with IFN-β. Similarly, another study did not find differences between endogenous type I IFN signature and disease course in MS treated patients even though the patients on the high IFN signature group showed weaker biologic response within the first treatment month [22].

These studies provide evidence of the role of baseline characteristics as determinants of the treatment response. The data presented in this study support that role and indicate that MxA may be a useful biomarker of IFN-β response in naïve relapsing-remitting MS patients. Patients with MxA levels under the threshold take longer to relapse and to increase by one point on the EDSS scale, likely because a less activated IFN pathway have greater ability to be stimulated when IFN-β treatment is started. Nonetheless, these findings should be validated in other MS cohorts. Our population only included treatment-naïve relapsing-remitting MS cases. The predictive performance of MxA should be tested in other forms of MS, such as the secondary progressive type, and in clinically isolated syndromes, and it would be interesting to see if there are differences between treatment-naïve and previously treated patients. Another issue to resolve in MxA mRNA measurement is the considerable variability between laboratories; hence, standardization of the technique is needed.

MxA after one year of treatment was studied in order to evaluate the bioavailability of IFN-β. One of the main causes of IFN bioavailability reduction is the development of NAbs that generally appear after 6-18 months of treatment [14], [15], [27]. In our study, only 18 patients failed in MxA induction after 12 months. Absence of MxA induction at month 12 was not related with baseline MxA levels. This suggests that, in our population, baseline MxA expression couldn't predict which patients were going to develop NAbs (evaluated through the absence of MxA induction at month 12). Therefore, the appearance of NAbs would be better related to the immunogenicity of the preparation, dosing frequency and route of administration [28] than to the baseline MxA levels. The survival analyses to study time to next relapse and to progression of disability didn't show differences between the induced and non-induced patients. Presence of NAbs may explain treatment failure after one year of treatment. Other biological mechanisms, such as the presence of soluble IFN receptors, could explain early treatment failure when NAbs are absent.

Over the last decades, IFN-β has been one of the most widely used treatments for MS. New therapies with better efficacy results, but also greater potential side effects, are now emerging. The development of biomarkers to decide whether one or another treatment is the most appropriate for each individual patient has become one of the principal objectives during the last years. Baseline MxA status had a positive predictive value of 0.77 and a negative predictive value of around 0.65 using the cut-off defined in this study. In conclusion, our results suggest that baseline MxA mRNA levels may be useful for predicting whether patients will respond or not to IFN-β, and this capability could be clinically useful for deciding on the most appropriate therapy option.
